# Generative AI as an External Cognitive Tool for Developing Creative Intelligence in Visual Design: A Mixed-Methods Randomized Study Using Cognitive Load Indicators and Motivational Modeling

**DOI:** 10.3390/jintelligence14040065

**Published:** 2026-04-14

**Authors:** Ziyang Huang, Jiajia Zhao, Xuan Fu

**Affiliations:** Department of Communication Design, College of Design, Graduate School, Hanyang University, Seoul 04763, Republic of Korea; pumpkin0402@hanyang.ac.kr (Z.H.); zhaojia31531@hanyang.ac.kr (J.Z.)

**Keywords:** generative AI, creative intelligence, domain-specific intelligence, cognitive load, learning motivation, psychometric modeling, expert-rated performance, PLS-SEM, human–AI co-creation

## Abstract

Generative artificial intelligence (GenAI) is rapidly transforming design education by enabling new forms of human–AI collaborative learning. However, how GenAI relates to cognitive and motivational processes in design learning contexts remains insufficiently understood. This study examines whether integrating GenAI into visual design instruction is associated with improvements in domain-specific creative performance and explores the relationships among cognitive load, learning motivation, and learning outcomes. A six-week randomized instructional experiment was conducted with 120 undergraduate students majoring in visual communication design. Creative performance was evaluated through blind expert ratings, and the relationships among key variables were analyzed using Partial Least Squares Structural Equation Modeling (PLS-SEM). The results show that GenAI-integrated instruction is associated with higher levels of learning motivation, engagement, and expert-rated creative performance compared with traditional instruction, whereas cognitive-load indicators show comparatively limited predictive strength within the overall model. In addition, Integrated Teaching Alignment (ITA) significantly moderates the relationship between perceived relevance and learning satisfaction. These findings suggest that GenAI may function as an external cognitive support tool, with learning outcomes appearing to be associated with motivational and instructional factors, while cognitive-load indicators show comparatively limited associations within this instructional context.

## 1. Introduction

In recent years, generative artificial intelligence (GenAI) has developed rapidly, and its potential applications in art and design education have become increasingly evident. With its ability to generate images, understand semantic information, and produce content efficiently, GenAI is gradually reshaping teaching models and knowledge structures in design education (Giannakos et al., 2025). Compared with traditional systems that rely on hand drawing and software-based technical skills, GenAI enables learners to complete complex visual generation through natural language, greatly expanding the expressive space of design thinking. However, this transformation also brings new challenges: how can learners maintain creative thinking when technology is deeply involved in the process, and how should teachers redefine their roles in a “human–AI co-creation” classroom? These questions have become central issues in the ongoing transformation of design education.

Research in educational psychology and cognitive science shows that learning outcomes depend not only on tool efficiency but also on learners’ cognitive load and motivation. To better understand learning mechanisms in AI-supported environments, this study draws on established perspectives from learning psychology, including cognitive load theory (CLT), which highlights that an appropriate balance of intrinsic load (IL), extraneous load (EL), and germane load (GL) can improve learning efficiency, and the ARCS Motivation Model, which suggests that attention (AT), relevance (RE), confidence (CF), and satisfaction (SA) are key drivers of learning motivation. With the introduction of GenAI, learners’ cognitive processing and motivational mechanisms may change, suggesting that traditional instructional models should be re-examined in AI-supported learning contexts.

Against this background, this study examines the impact of GenAI-integrated instruction on learning performance using a structured instructional design and established theoretical frameworks. In line with the United Nations Sustainable Development Goal 4 (SDG 4), it explores how GenAI-supported instructional models can improve educational quality and support innovation-oriented learning environments in design education. By integrating learning theories with AI-supported instructional practices, the study provides theoretical and practical insights for developing more inclusive and sustainable design education systems.

Building on the theoretical perspectives discussed above, this study examines the integration of generative AI in visual design education. In this study, generative AI is conceptualized as an external cognitive tool, defined as a technological system that supports learners’ thinking by partially externalizing cognitive processes such as idea generation, visualization, and iterative exploration into an AI-assisted environment. In this sense, GenAI functions not only as a production tool but also as a cognitive scaffold that allows learners to offload certain cognitive tasks to an external system.

By integrating GenAI with the five stages of design thinking, this study proposes a systematic instructional framework based on a “task-chain + tool-chain” structure to support human–AI collaborative learning. Using a visual design course as the empirical context, a six-week illustration design experiment was conducted to examine the instructional effects of GenAI-integrated teaching. The study addresses the following research questions:

RQ1. How is GenAI-integrated instruction associated with changes in students’ cognitive load and learning motivation in visual design courses?

RQ2. Can CLT and the ARCS model effectively reveal the mechanisms linking cognition, motivation, and learning performance in a GenAI instructional environment?

RQ3. In GenAI -supported instructional settings, does perceived Integrated Teaching Alignment (ITA) moderate the relationships between learning motivation and both learning satisfaction and design outcomes?

RQ4. Compared with traditional instructional models, does GenAI-integrated instruction lead to differences in learning outcomes and creative performance?

Despite growing interest in GenAI in education, existing studies mainly focus on tool effectiveness and pedagogical applications, while the cognitive and motivational mechanisms underlying AI-supported learning remain underexplored, particularly in design education. In addition, empirical evidence based on randomized instructional designs in design education is still limited.

To address this gap, this study develops a structural equation model based on CLT and the ARCS model and examines the instructional effects of GenAI-integrated teaching through a six-week visual design course experiment. A mixed-methods approach combining questionnaires, expert evaluations, and interviews is used to analyze learning outcomes and motivational processes. In addition, Integrated Teaching Alignment (ITA) is introduced as a moderating construct, referring to learners’ perceived coordination among instructional tasks, AI tool support, and learning processes in AI-supported environments. By integrating cognitive load, learning motivation, and instructional alignment within a unified analytical framework, this study provides empirical evidence on how GenAI-integrated instruction influences cognitive processes, motivational dynamics, and creative performance in visual design education.

## 2. Literature Review

### 2.1. Current Applications of Generative AI in Design Education

In recent years, research on GenAI in the field of education has deepened considerably, and its pedagogical potential and cognitive challenges within design education have become prominent topics of academic inquiry. Overall, numerous studies have identified GenAI as a major driving force for instructional innovation; however, its potential drawbacks—such as the weakening of originality, ethical concerns, and technology dependence—have also been repeatedly reported (Zhang et al., 2025). The current research focus has gradually shifted from single-technology evaluation toward a more integrated exploration of learning performance, motivational regulation, and human–AI collaboration mechanisms, offering new perspectives for the paradigm transformation of design education.

At the theoretical level, systematic reviews have identified several key applications of artificial intelligence in education, including feedback systems, adaptive learning, and immersive learning environments, which provide an important theoretical basis for understanding the structural role of GenAI in design education (Zhai et al., 2021). Further research has analyzed the functional mechanisms of GenAI across four stages of the design process—creative ideation, design generation, assisted suggestion, and evaluative feedback—and proposed an evolutionary model of AI integration in design education, offering a structured framework for subsequent studies (Wu et al., 2025). From a broader perspective, recent studies have also examined the opportunities and challenges associated with GenAI in education. Although GenAI can enhance learning processes, it may also introduce ethical concerns and potential imbalances within the educational ecosystem, highlighting the need for a human-centered AI education framework that supports sustainable development (Giannakos et al., 2025).

In terms of practical instructional applications, recent studies have examined the integration of GenAI into design education from multiple perspectives. A systematic evaluation of GenAI-supported teaching in interior design education, based on the UTAUT model and the AHP method, showed that AI tools can effectively support instructional decision-making and learning processes (Liu et al., 2025). Experimental studies in graphic design courses using tools such as Midjourney and DALL·E further demonstrated that GenAI can reshape course objectives and learning processes (Hwang & Wu, 2025). Empirical observations across multiple university visual design courses also indicate that the introduction of GenAI is reshaping course structures and lecturer–student interaction patterns, promoting a shift from teacher-centered instruction toward a more learner-centered and open learning ecosystem (K. Wang et al., 2025).

Overall, existing studies generally affirm the learning-enhancing effects of GenAI in design education; however, issues such as ethical risks, assessment reliability, and the weakening of originality still require further investigation. Research on GenAI has gradually shifted from a focus on “tool efficiency” to an integrated mechanism encompassing cognitive load, learning motivation, and learning outcomes. Nevertheless, systematic empirical validation of the underlying cognitive and motivational pathways remains insufficient.

### 2.2. Application of Cognitive Load Theory in Art and Design Education

Cognitive load theory (CLT) was first proposed to explain how the limited capacity of working memory constrains learning during problem solving (Sweller, 1988). The theory was later refined by distinguishing three types of cognitive load—intrinsic (IL), extraneous (EL), and germane (GL)—and highlighting their implications for instructional design (Sweller et al., 1998). Subsequent studies further extended CLT to technology-enhanced and multimedia learning environments, suggesting that appropriate regulation of these loads can improve learning efficiency (Sweller et al., 2011). With the increasing use of artificial intelligence and immersive technologies in art and design education, recent research has begun to explore how technological and instructional interventions can dynamically regulate cognitive load and support creative performance.

Early studies have shown that instructional design can significantly influence cognitive load. Moderately disfluent learning materials may increase extraneous load but can also improve comprehension and memory by introducing appropriate learning challenges (İliç & Akbulut, 2019). In immersive learning contexts, animation-supported virtual disassembly can enhance conceptual understanding and learning performance, whereas augmented reality (AR) may increase extraneous load due to information complexity (Kearney et al., 2022). In addition, emotional factors are closely related to cognitive load regulation. Although gamified learning can reduce anxiety and increase motivation, competitive elements may simultaneously raise cognitive and emotional load (Y. Chen et al., 2022). These findings suggest that effective instructional design should balance learning motivation and cognitive load.

In AI-assisted instruction, CLT research has increasingly focused on human–AI collaboration and cognitive offloading. AI may support design thinking by reducing extraneous load and increasing germane load in dual-path teaching contexts (Dong et al., 2025). Similarly, embodied GenAI systems can improve immersion and autonomy while reducing overall cognitive load in human–AI co-learning (Nguyen et al., 2025). In collaborative learning settings, optimized task structures and scaffolded support have also been shown to reduce extraneous load and promote knowledge construction (X. Du et al., 2023). Together, these findings suggest that in GenAI-integrated learning environments, lecturers need to act as both cognitive regulators and thinking facilitators.

In summary, technological intervention does not necessarily reduce EL; its effectiveness depends on the alignment between information presentation and task complexity. At the same time, emotional and motivational factors interact dynamically with cognitive load. It should also be noted that the measurement of cognitive load remains a topic of discussion in educational research, particularly regarding subjective self-report and objective indicators, as well as the dimensional validity of load constructs. Accordingly, the present study considers cognitive load as an important mediating factor influencing learning performance in GenAI-based instruction, aiming to better understand the relationships among cognitive regulation, creative thinking, and learning outcomes.

### 2.3. The ARCS Motivation Model and Studies on Learning Motivation Mechanisms

While CLT explains cognitive processes in GenAI-based instruction, the ARCS model focuses on learning motivation. The ARCS framework identifies four dimensions of learner motivation: attention (AT), relevance (RE), confidence (CF), and satisfaction (SA) (Keller, 1987, 2009). It emphasizes regulating learners’ motivational states through instructional design to support sustained engagement and participation. With the increasing use of artificial intelligence, virtual reality, and gamified learning, the ARCS model has become an important framework for analyzing motivational dynamics in GenAI–design thinking learning environments.

In higher education, the ARCS model has been validated as an effective framework for analyzing learning motivation in blended learning environments (Ma & Lee, 2021). Research in digital game-based learning further shows that ARCS-based instructional design can significantly enhance learner motivation across multiple dimensions (Camacho-Sánchez et al., 2024). Similar effects have also been observed in GenAI-integrated instruction, where AI-generated feedback and visual content can increase attention, while iterative creation processes support learners’ confidence and satisfaction.

In technology-enhanced learning environments, augmented reality (AR) games have been shown to increase learners’ attention and satisfaction, while having limited effects on relevance and confidence (Sdravopoulou et al., 2021). Longitudinal research on gamified learning further shows that learner motivation may decline mid-semester but recover toward the end, indicating a nonlinear motivational pattern (Van Roy & Zaman, 2018). Studies combining the ARCS model with goal orientation theory also suggest that higher model congruence can reduce learning anxiety and enhance motivation in virtual reality simulation environments (Lin et al., 2025). These findings indicate that alignment between task design and learning goals plays an important role in sustaining motivation.

In AI-assisted instruction, generative AI systems such as ChatGPT can provide personalized feedback and real-time guidance, improving learner motivation, self-efficacy, and academic performance (J. Chen et al., 2025). Research integrating the ARCS model and constructivist learning theory further shows that instructional quality and learning environments can influence learning outcomes through motivational enhancement (Pang et al., 2025). These findings are consistent with the ITA concept proposed in this study, which emphasizes the joint role of lecturer design, AI support, and learner perception in shaping motivational pathways.

According to existing research, the sequence of visual generation supporting Attention (A), task relevance corresponding to Relevance (R), instant feedback contributing to Confidence (C), and outcome satisfaction related to Satisfaction (S) constitutes a typical motivational pathway in GenAI-mediated design learning. However, the interactive mechanisms and quantifiable indicators linking GenAI-supported instructional processes with learning motivation have yet to be systematically validated. Therefore, grounded in CLT and the ARCS model, the present study introduces ITA as a moderating construct in the proposed cognition–motivation–learning performance model, which is empirically examined through questionnaire surveys and PLS-SEM analysis.

## 3. Research Design and Methods

### 3.1. Research Methodology

**Figure 1:**
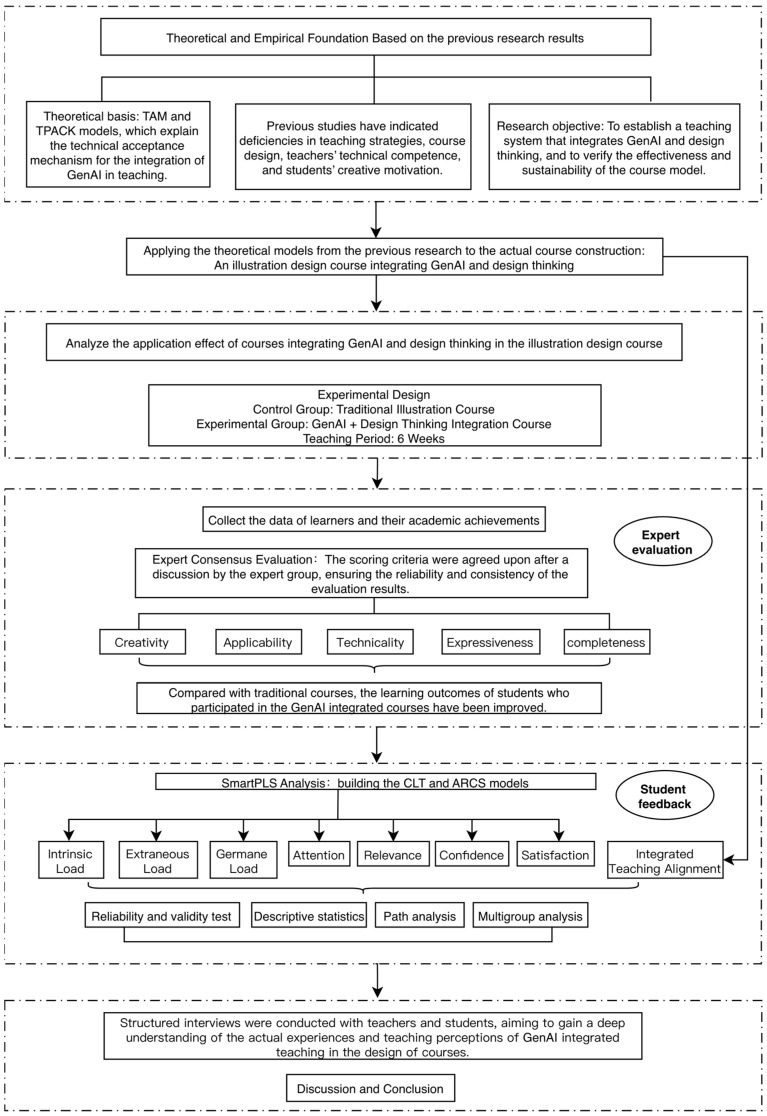
Research process.

The overall procedure of this study is shown in Figure 1. Based on prior research and the theoretical framework, the research objectives were clarified, and a six-week illustration course experiment was designed. The control group received traditional instruction, whereas the experimental group adopted a GenAI- and design-thinking-integrated approach.

Student learning data and design outcomes were collected and combined with expert evaluations to assess instructional effects. SmartPLS was used to validate the path model based on CLT and the ARCS model, including reliability and validity testing, descriptive statistics, path estimation, and multi-group comparisons to examine model robustness and group differences. PLS-SEM was selected because it performs well with relatively small sample sizes and complex models involving multiple latent constructs.

Finally, structured interviews with lecturers and students were conducted to capture emotional, cognitive, and interactional changes that are difficult to identify through quantitative analysis. By integrating quantitative and qualitative results, the effectiveness of the instructional model was evaluated, providing empirical and theoretical support for future course optimization and instructional design.

### 3.2. Construction of the GenAI-Integrated Visual Design Course

**Figure 2:**
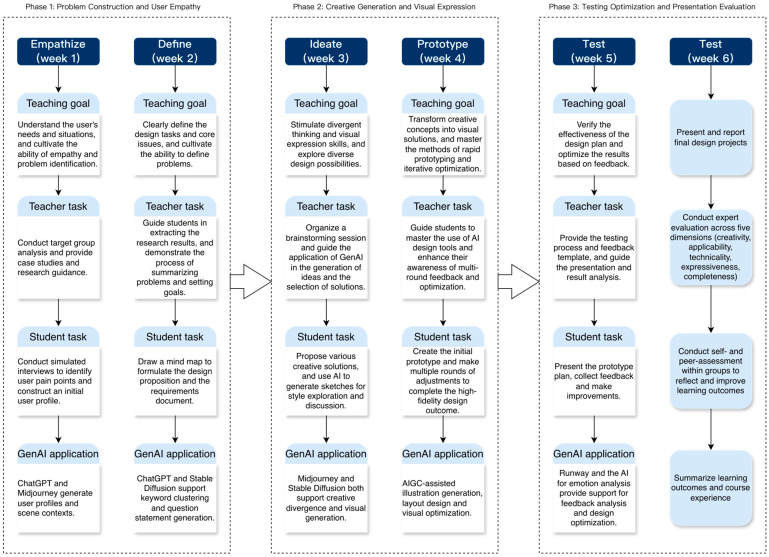
Course outline.

Based on prior research findings, this study integrates design thinking theory, the TPACK model, and a composite perspective of CLT and the ARCS model to construct a GenAI-integrated instructional course within a “task-chain + tool-chain” framework. The course design draws on the five stages of design thinking—Empathize, Define, Ideate, Prototype, and Test—as the conceptual backbone of the instructional process. To better fit the classroom context, the instructional procedure is operationalized into six instructional stages, including an additional course summary and reflection stage. For clarity of presentation, these six stages are further grouped into three broader instructional phases in Figure 2, illustrating how GenAI tools are embedded throughout the instructional process and providing a structured context for the subsequent analysis of learning mechanisms.

As shown in Figure 2, the GenAI-integrated design thinking course consists of six stages, each with specific learning tasks and GenAI support. The process moves from user empathy and problem definition to ideation, prototyping, testing, and reflection. Throughout the course, GenAI supports information gathering, idea generation, visual production, and feedback analysis (Lai et al., 2023). This structure reflects a human–AI collaborative learning model in which lecturers guide the process, students engage in task-based practice, and AI tools function as cognitive and creative assistants. The framework provides a practical basis for the subsequent instructional experiment.

### 3.3. Research Hypotheses

**Figure 3:**
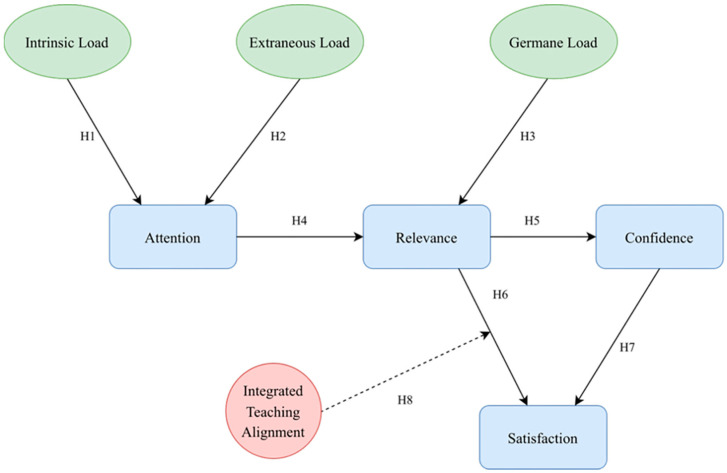
Research hypothesis.

This study develops a structural equation model (SEM) based on CLT, ARCS, the TPACK framework, and constructivist learning theory, and proposes the following research hypotheses (see Figure 3). It should be noted that these hypotheses describe the theoretically expected relationships, and their specific functional forms will be further examined through empirical analysis.

1. Cognitive Load and Learning Process.

According to CLT, learners are mainly influenced by IL, EL, and GL during the learning process (C. C. Wang et al., 2022). Prior research suggests that excessively high IL and EL may consume learners’ limited cognitive resources, whereas GL facilitates knowledge construction and deep processing (Minkley et al., 2021). Therefore, this study proposes the following hypotheses:

**H1:**  *There is a negative relationship between IL and AT.*

**H2:**  *There is a negative relationship between EL and AT.*

**H3:**  *There is a positive relationship between GL and RE.*

2. The Pathway between Attention and Relevance

In the ARCS model, AT is considered the starting point of learning motivation, while RE reflects the alignment between learning content and learners’ goals (Li, 2022). Previous research suggests that sustained attention helps strengthen the connection between learning content and personal goals (Strayer et al., 2024). Therefore, this study proposes the following:

**H4:**  *There is a positive relationship between AT and RE.*

3. Relevance, Confidence, and Satisfaction

Motivational theory further suggests that when learners perceive a high level of RE between course content and their own goals, their CF and overall learning SA are likely to increase accordingly (Y. Wang & Song, 2022). Based on this, the present study proposes the following hypotheses:

**H5:**  *There is a positive relationship between RE and CF.*

**H6:**  *There is a positive relationship between RE and SA.*

**H7:**  *There is a positive relationship between CF and SA.*

4. The Moderating Role of Integrated Teaching Alignment (ITA)

This study introduces ITA as a moderating variable to describe the systemic alignment among lecturer instructional design, GenAI tool support, and learners’ perceptions within GenAI-supported instructional contexts. This variable is conceptually derived from the TPACK framework and constructivist learning theory, and reflects learners’ overall perception of the degree of coordination among lecturer guidance, GenAI tool use, and the design thinking process. Based on this, the present study proposes the following:

**H8:**  *ITA exerts a positive moderating effect on the relationship between RE and SA, such that the positive relationship between RE and SA is stronger when the perceived level of ITA is higher.*

### 3.4. Instructional Experiment Design

This study recruited 120 s-year undergraduate students majoring in Visual Communication Design at College X as the research participants. At this stage, the students had already developed relatively stable visual-modeling abilities and digital-drawing skills, making them suitable for participating in an illustration course supported by GenAI. To ensure the internal validity and interpretability of the experimental results, the study was systematically designed and reported across four dimensions: grouping method, baseline equivalence, intervention consistency, and threat control.

#### 3.4.1. Participant Recruitment and Group Randomization

All participants were second-year students from the same major and cohort who were enrolled in the Illustration Design course during the same semester and voluntarily participated in the study. Participants were randomly assigned at the individual level into an experimental group and a control group, with 60 students in each group, and the two groups were taught in separate classes. Before the course began, a computer randomization function was used to randomly allocate student ID numbers to either group, generating the final grouping list. Group assignments were then kept unchanged throughout the six-week instructional period.

#### 3.4.2. Pre-Intervention Baseline Measurement and Group Equivalence Testing

Before the formal instructional intervention, this study conducted a systematic baseline measurement (pretest) to confirm the comparability of the experimental and control groups at the starting point of the research and to reduce the potential interference of prior technical experience, professional competence, and learning motivation on subsequent learning performance. The baseline measurement focused on four key dimensions, corresponding to GenAI technical experience, tool operation proficiency, professional design foundation, and learning motivation level.

(1) The baseline dimension of GenAI usage experience was used to control differences in participants’ prior exposure to generative AI technology. Specifically, students were asked whether they had used mainstream GenAI tools (such as Midjourney, DALL·E, ChatGPT-4, etc.) and to report their frequency of use. This indicator was mainly intended to identify differences in students’ level of technical exposure, so as to avoid confounding effects on subsequent learning efficiency and cognitive load caused by whether or not students had previously used GenAI.

(2) For the baseline dimension of GenAI/prompt-operation proficiency, beyond distinguishing whether students used GenAI or not, this study further measured students’ actual operational proficiency with GenAI tools, with particular attention to self-evaluated ability in writing and refining prompts. This dimension was used to control differences in operational competence and to prevent uneven tool proficiency from influencing students’ performance and learning experience during the creative-generation phase.

(3) For the baseline dimension of design foundation competence, a unified rapid concept-development or sketching task (20–30 min) was arranged before the course began to control initial differences in professional ability. Students were required to complete a basic visual-concept task under the same time and conditions. The outputs were evaluated by two course instructors participating in the experiment using a unified scoring rubric. This dimension was used to exclude the potential interference of students’ prior design ability on the experimental results.

(4) For the baseline of learning motivation (ARCS-related dimensions), given that learning motivation may have an important impact on the effectiveness of the instructional intervention, a brief pretest of students’ motivational level was conducted before the course. Dimensions in the ARCS framework that are closely related to course engagement (such as perceived relevance and task confidence) were selected for measurement. This dimension was used to control differences between the two groups in their initial learning attitudes and willingness to engage.

These four baseline dimensions systematically controlled key confounding factors that may affect the effectiveness of the instructional intervention from the perspectives of technical experience, operational ability, professional skills, and learning motivation. Independent-sample *t*-tests (for continuous variables) and chi-square tests (for categorical variables) were used to compare the baseline data between groups, and effect sizes (Cohen’s d or φ) were also reported. The results indicated that there were no significant differences between the experimental and control groups on any baseline indicator (*p* > 0.05, with all effect sizes within the small-effect range), demonstrating good equivalence between the two groups at the beginning of the study. This provided a reliable foundation for the subsequent comparison and interpretation of instructional outcomes (see Table 1).

#### 3.4.3. Intervention Implementation and Fidelity Control

To minimize systematic bias caused by teacher effects and differences in instructional resources, strict fidelity control was applied during the intervention. Both groups were taught by the same team of lecturers, with identical teaching weeks and total class hours. The course topics, assignment requirements, lecture content, and evaluation checkpoints were also kept consistent, and both groups completed their tasks in the same instructional environment with the same equipment. The only difference lay in the teaching strategy and the way GenAI was integrated: the control group followed a traditional illustration-teaching process, using GenAI tools only sporadically during sketching or material-preparation stages; in contrast, the experimental group adopted a “GenAI-embedded instructional model” based on the five stages of design thinking, setting explicit tool-intervention points across the Empathize–Define–Ideate–Prototype–Test stages to form a closed loop structure of “task chain + tool chain” (see Table 2). By comparing the learning performance of the two groups, the effectiveness of a systematic GenAI-integrated instructional model can be verified (Weng et al., 2024).

#### 3.4.4. Identification and Control of Threats to Internal Validity

In response to potential threats to internal validity, the following control strategies were adopted:

(1) Teacher effect: The same lecturers, a unified teaching plan, and consistent evaluation criteria were applied to both groups to minimize the influence of instructor differences.
(2) Time effect: Both groups completed the instructional experiment during the same semester and within the same six-week period to avoid systematic differences caused by external timing factors.
(3) Initial motivation difference: A motivation pretest was conducted before the course, followed by equivalence testing. Where necessary, baseline motivational variables were treated as covariates in subsequent statistical analyses.
(4) Novelty effect and tool-familiarity bias: Before the formal course began, both groups received the same introductory briefing and operational guidance on GenAI tools (entry-level training), which served as a unified starting point. In addition, phased tasks and iterative feedback were used in the experimental group to reduce volatility caused by short-term excitement.

Through this design, the study ensured inter-group equivalence at the outset and controlled key confounding factors during the intervention, thereby improving the internal validity and reproducibility of the findings. By comparing the learning performance of the two groups, the effectiveness of the systematic GenAI-integrated instructional model can be evaluated. All students participated with informed consent, and data collection and analysis followed academic ethics and privacy protection requirements. After the experiment, the control group received a supplementary course with the same integrated instructional content to ensure educational fairness (B. Du & Guo, 2023).

### 3.5. Data Collection Methods

#### 3.5.1. Expert Evaluation Procedure and Consistency Control

To verify the external validity of learning outcomes in both groups, six experts with teaching and industry experience in illustration and visual communication were invited to conduct a multidimensional evaluation of students’ final works. The evaluation was carried out in an on-site panel session, during which all experts assessed all works within the same session and time window (Zhao et al., 2024). Before scoring, the researchers provided a unified scoring guideline and rating rubric, and conducted a brief orientation to align evaluation procedures and criteria across dimensions, thereby ensuring consistency in evaluative understanding.

To reduce potential bias from group information and order effects, all works were anonymized prior to evaluation by removing student names, group identifiers, and other identifiable information. The works were then standardized into presentation materials with a consistent structure, including final images, brief design statements, and key process summaries. Subsequently, a computer-based randomization function was used to determine the presentation order and assign identification codes, and experts independently evaluated the works following this randomized sequence.

To control for the potential efficiency advantage of GenAI and to avoid confounding speed with instructional effects, this study standardized time-on-task for both groups at the course design level. The experimental and control groups completed their tasks within the same number of teaching weeks, identical in-class hours, and identical assignment windows, and submitted their final works at the same deadline. The only difference between the groups was whether GenAI was used for generation and iteration under the same time constraints. This design ensured that differences in work quality reflected variations in instructional structure and learning processes rather than differences in time investment or submission conditions.

The evaluation objects were the final group projects completed by students in the experimental and control groups during the course (24 projects in total), and scoring was conducted on a 100-point scale. The evaluation dimensions were jointly developed by experts based on the course objectives and task characteristics, covering five aspects: creative performance, application relevance, production quality, esthetic communication, and overall logic (Shyr et al., 2019). Before formal scoring, the experts conducted a calibration exercise using sample works to align their understanding of the rating scale. For each project, the mean score across the five dimensions was used as the overall evaluation result for subsequent between-group comparisons and model validation. Expert ratings (outcome evaluation), together with questionnaire-based process data, formed a multidimensional validation framework encompassing instructional process, learning experience, and outcome quality (see Table 3).

#### 3.5.2. Questionnaire Design Based on CLT and ARCS Models

To analyze learners’ cognitive and psychological changes under different teaching models, a questionnaire was developed based on the CLT and ARCS frameworks to measure variations in cognitive load, learning motivation, and integrated perception (as shown in Table 4). The questionnaire adopted a three-dimensional structure of Cognition–Motivation–Perception, comprising eight latent variables and 24 items, measured on a five-point Likert scale (1–5). The scale was adapted from the seminal works of Sweller and Keller, and localized to fit the context of commercial illustration instruction. The questionnaire was distributed and collected at the end of the course to ensure data completeness and comparability.

This process-oriented data systematically reveals the relationships among cognitive load, learning motivation, and perceived instructional alignment, providing empirical support for validating the effectiveness of the GenAI-integrated teaching model. Together with the expert evaluation of student works, it forms a complementary multidimensional assessment framework for comprehensive evaluation.

#### 3.5.3. Design and Implementation of Structured Qualitative Interviews

To gain a deeper understanding of the actual experience of GenAI-integrated instruction, this study conducted structured interviews after the course, involving 12 respondents in total, including two lecturers and 10 students from the experimental group. The interviews focused on themes such as GenAI application experience, cognitive changes, motivational regulation, and instructional integration. Through thematic analysis, the study supplemented the quantitative findings by capturing changes in learning and teaching perceptions that are difficult to reflect through numerical data alone, revealing issues in the application of the model and proposing directions for future course optimization (see Table 5 and Table 6).

The interview data were analyzed using a consensus-based thematic coding approach, following an inductive thematic analysis procedure. Two researchers independently reviewed the interview transcripts and generated initial codes based on key themes related to cognition, motivation, and instructional integration. The coding results were then compared, and discrepancies were resolved through discussion until an agreement was reached on the final coding framework and thematic categories. Formal inter-coder reliability statistics were not calculated, as the qualitative component was intended to provide supplementary interpretive support for the quantitative findings rather than to establish an independent coding framework. This approach is consistent with the exploratory and supportive role of the qualitative analysis in this study.

## 4. Research Results and Analysis

After the instructional experiment was completed, the study proceeded to the data-analysis stage. Questionnaire data from the experimental and control groups were used to conduct descriptive statistics and reliability–validity testing on core variables such as cognitive load and learning motivation. This process was intended to verify the structural reliability of the scales and the suitability of the data for analysis, thereby providing a sound basis for subsequent difference testing and structural equation modeling.

### 4.1. Descriptive Statistical Results

Table 7 summarizes the means and standard deviations of the eight latent variables for the experimental and control groups. Overall, the experimental group shows higher mean levels on most dimensions, preliminarily indicating distributional differences in learning experience under GenAI-integrated instructional conditions. The results in this section are purely descriptive and are intended to present overall distribution characteristics and between-group trends. The statistical significance of these differences and the relationships among variables still require verification through subsequent structural modeling.

In terms of cognitive load, the experimental group reported higher subjective ratings on IL (M = 3.700, SD = 1.211) and EL (M = 3.778, SD = 1.080) than the control group, with IL (M = 3.000, SD = 1.111) and EL (M = 2.822, SD = 1.009). Meanwhile, the experimental group also scored higher on GL (M = 3.744, SD = 1.196) compared with the control group (M = 3.133, SD = 1.237). These results suggest that learners in the experimental group perceived more complex cognitive and operational experiences during task completion rather than directly implying a causal influence of cognitive load on learning outcomes.

In terms of learning motivation, the experimental group reported higher mean levels of AT, RE, CF, and SA than the control group, with the increases in RE and AT being relatively more pronounced. By contrast, the between-group difference in CF was smaller, implying that changes in learning confidence may be shaped by multiple interacting factors, and that its functional mechanism requires further verification through structural modeling.

In addition, the experimental group reported a higher mean on ITA (M = 3.844, SD = 1.038) than the control group (M = 3.544, SD = 1.255), indicating a more positive overall perception of course organization, lecturer support, and tool alignment. With respect to dispersion, the standard deviations varied across dimensions and groups, reflecting individual differences in learners’ subjective perceptions of new tools and collaborative instructional structures under identical teaching conditions. Overall, the descriptive results show a general tendency toward higher subjective cognitive perceptions and motivational experiences in the experimental group, providing a preliminary empirical basis for subsequent PLS-SEM path testing and between-group comparisons.

### 4.2. Measurement Model Validation (Reliability and Validity Analysis)

Before analyzing the structural model, the reliability and validity of the measurement model were first examined to ensure that the measurement quality of each latent variable met the basic requirements for subsequent structural equation analysis. The evaluation of the measurement model focused on three aspects: internal consistency reliability, convergent validity, and discriminant validity.

Internal consistency reliability was assessed using Cronbach’s alpha and Composite Reliability (CR). As shown in Table 8, the Cronbach’s alpha values for all constructs ranged from 0.779 to 0.969, all exceeding the recommended threshold of 0.70 (Taber, 2018). Meanwhile, the CR values for all constructs ranged from 0.870 to 0.973, indicating good internal consistency and stability of the scales (Raykov & Marcoulides, 2019). In addition, the factor loadings of all measurement items exceeded the recommended threshold of 0.70, indicating satisfactory indicator reliability. Although the Cronbach’s alpha value for IL reached 0.969, which may suggest potential item redundancy, it also reflects the high internal consistency among the measurement items.

Convergent validity was assessed using Average Variance Extracted (AVE). The results showed that the AVE values for all constructs were greater than 0.50, ranging from 0.642 to 0.924, indicating that each latent variable could effectively explain the variance of its measurement indicators and that the measurement model demonstrated good convergent validity (Fornell & Larcker, 1981).

Discriminant validity was examined using the Heterotrait–Monotrait Ratio (HTMT) method. As shown in Table 9, the HTMT results indicated that all construct pairs had HTMT values below the conservative threshold of 0.85, suggesting good discriminant validity among the constructs and supporting the discriminant validity of the measurement model (Henseler et al., 2015).

Overall, the measurement model in this study met the standards for structural equation modeling in terms of internal consistency reliability, convergent validity, and discriminant validity, thereby providing a reliable foundation for subsequent path analysis and hypothesis testing in the structural model.

In addition, Harman’s single-factor test was conducted to examine potential common method bias resulting from the self-reported questionnaire data. The results indicate that the first unrotated factor accounted for less than 50% of the total variance, suggesting that common method bias is unlikely to pose a serious threat to the validity of the results.

### 4.3. Structural Model Results and Path Analysis

**Figure 4:**
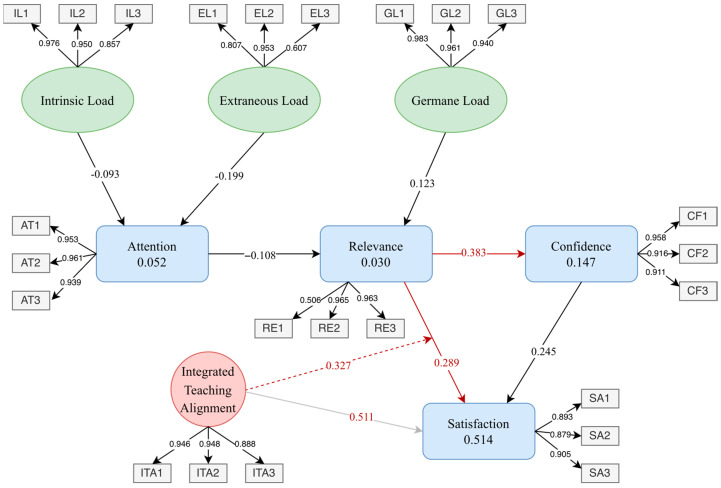
Path analysis diagram. Note: Red solid lines indicate hypothesized paths that are statistically significant; red dashed lines indicate statistically significant paths that are not included in the hypotheses; black/gray lines indicate non-significant paths.

To test the research hypotheses, this study employed Partial Least Squares Structural Equation Modeling (PLS-SEM) to conduct a path analysis of the theoretical model (see Figure 4). The model-fit indices indicated good overall fit (SRMR = 0.063, NFI = 0.912), and the variance inflation factors (VIFs) for all paths were below five, indicating no substantial multicollinearity and demonstrating that the structural model provided a reliable basis for estimation (Hair et al., 2021).

In the structural path results, ITA showed a significant positive effect on learning satisfaction (SA) (β = 0.511, t = 3.462, *p* = 0.001), with a relatively large effect size (f^2^ = 0.495). In addition, ITA exhibited a significant positive moderating effect on the RE → SA path (β = 0.327, t = 2.043, *p* < 0.05, f^2^ = 0.191), supporting Hypothesis H8.

Regarding the relationships among motivational variables, course relevance (RE) had a significant positive influence on learning confidence (CF) (β = 0.383, t = 2.089, *p* < 0.05, f^2^ = 0.172), supporting Hypothesis H5. However, the direct path from RE to SA (β = 0.289, *p* = 0.155) and the path from CF to SA (β = 0.245, *p* = 0.213) did not reach statistical significance.

For the cognitive load-related paths, the effects of intrinsic load (IL) on attention (AT) (β = −0.093), extraneous load (EL) on AT (β = −0.199), germane load (GL) on course relevance (RE) (β = 0.123), and AT on RE (β = −0.108) were all statistically non-significant.

In terms of explanatory power, the coefficient of determination for learning confidence (CF) was R^2^ = 0.147, while that for learning satisfaction (SA) was R^2^ = 0.514, indicating that the model demonstrates a moderate-to-high level of explanatory capability for learning satisfaction (Sarstedt et al., 2021).

### 4.4. Measurement Invariance Assessment (MICOM)

To enable valid comparisons between groups, measurement invariance was assessed prior to conducting multi-group analysis. To ensure the validity of subsequent multi-group analysis, measurement invariance across groups was assessed using the MICOM (Measurement Invariance of Composite Models) procedure.

First, configural invariance was established, as both groups used identical measurement items, data processing procedures, and model specifications.

Second, compositional invariance was evaluated using a permutation test. As shown in Table 10, compositional invariance was supported for all constructs.

Third, the equality of composite means and variances was examined. The results indicated no significant differences in composite variances. However, EL, IL, and RE showed significant differences in composite means and therefore did not meet the criteria for full measurement invariance. Accordingly, partial measurement invariance was established, which supports cautious and meaningful comparison of structural relationships across groups.

### 4.5. Multi-Group Comparison Results Between Experimental and Control Groups

**Figure 5:**
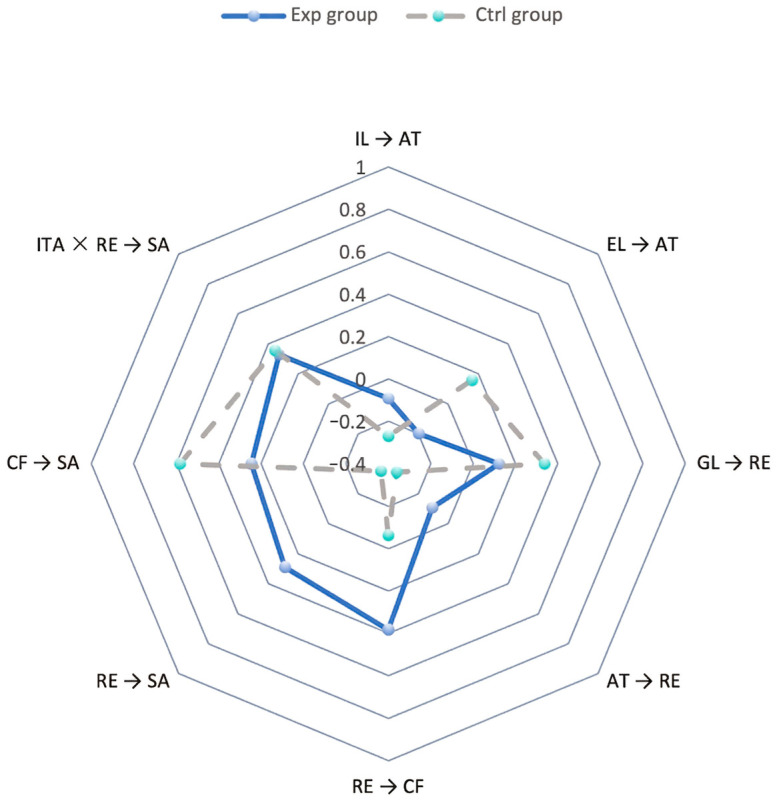
Multi-group Path Difference Data radar chart.

To further examine the impact of GenAI-integrated instruction on learning mechanisms, multi-group analysis (MGA) was conducted using PLS-SEM with a permutation test to compare path differences between the experimental and control groups. Based on the established partial measurement invariance using the MICOM procedure, comparisons of structural relationships across groups are considered valid. This analysis focuses on identifying statistically significant between-group differences, complementing the overall path results reported in Section 4.3. The results suggest that there are statistically significant differences between the two groups on several key paths (see Table 11 and Figure 5).

#### 4.5.1. Analysis of Direct Path Differences

The results of the multi-group analysis (MGA) showed that several structural paths differed significantly between the experimental and control groups (see Table 11). Specifically, the effect of GL → RE was significantly weaker in the experimental group than in the control group (difference = −0.214, *p* = 0.037). In addition, the effect of AT → RE also showed a significant between-group difference (difference = 0.236, *p* = 0.005), with opposite path directions observed across the two groups.

For the motivation- and outcome-related paths, the effect of RE → SA differed significantly between the two groups (difference = 0.641, *p* = 0.009). By contrast, although differences in direction or magnitude were observed for RE → CF, CF → SA, and ITA × RE → SA, these differences were not statistically significant and were therefore not supported by the MGA results.

Overall, the pattern of path differences suggests that certain relationships vary across instructional conditions, indicating context-dependent differences between the two groups.

#### 4.5.2. Analysis of Indirect Path Differences

To further compare differences in indirect effects between the experimental and control groups, a multi-group comparison of selected theoretically relevant indirect paths was conducted using MGA with permutation testing. The results are presented in Table 12. The focus of this analysis is to examine whether indirect effects differ across instructional contexts, rather than to infer their causal mechanisms.

As shown in Table 12, three indirect paths showed significant between-group differences: AT → RE → SA (*p* = 0.005), GL → RE → SA (*p* = 0.037), and IL → AT → RE → SA (*p* = 0.007). The remaining indirect paths did not show statistically significant differences (*p* > 0.05).

Because mediation analysis is not the central focus of this study, these findings are reported as supplementary evidence only and are not interpreted as stable mediation mechanisms. The observed differences should be interpreted with caution, as they primarily serve to provide contextual insights rather than definitive structural interpretations.

### 4.6. Expert Evaluation and Model Validation

To further examine the performance of GenAI-integrated instruction for students’ design outcomes, six experts with experience in design teaching and practice independently evaluated anonymized works in an on-site panel session using a unified scoring rubric. Discrepancies in scoring were resolved through iterative discussion until consensus was reached. The presentation order of the works was randomized using a computer-generated function. Both groups completed and submitted their works within the same course duration and under the same deadline to control for time-related factors.

It should be noted that the expert evaluation was conducted at the project level (*n* = 24), focusing on the quality of final design outcomes, whereas the SEM analysis was performed at the student level (*n* = 120), focusing on patterns observed in the learning process. These two analyses operate at different analytical levels and serve complementary roles within the research design. The aggregated expert evaluation results are presented in Table 13.

To statistically verify the differences observed in the expert evaluation results, an independent-sample *t*-test was conducted at the project level (*n* = 24). The results indicated that the experimental group achieved significantly higher expert evaluation scores (M = 86.58, SD = 1.84) than the control group (M = 79.94, SD = 2.98), t(22) = 6.57, *p* < 0.001. The effect size was large (Cohen’s d = 2.68), suggesting a substantial difference in expert-rated design outcomes between the two instructional conditions.

**Figure 6:**
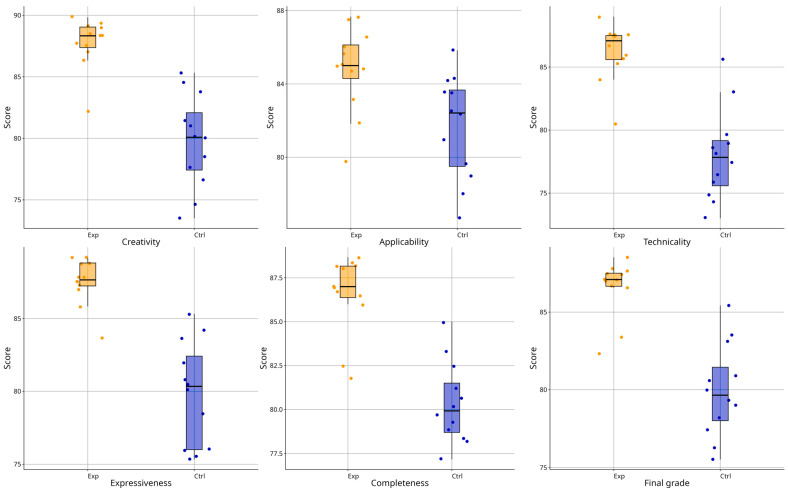
Comparison of design performance scores between experimental and control groups.

Figure 6 shows the distribution of expert evaluation scores for the experimental and control groups across five dimensions and the overall score. The boxplots present median values, interquartile ranges (IQRs), and potential outliers, while the overlaid scatter points represent individual ratings assigned by the six experts. Overall, the experimental group has higher median scores than the control group across all dimensions, with distributions shifted toward higher values. In the Technicality and Expressiveness dimensions, experimental group scores are mainly concentrated around 85–88, whereas control group scores cluster around 75–80, with little overlap between distributions. Similar patterns appear in the Creativity and Completeness dimensions, where the experimental group shows narrower IQRs, while the control group displays greater dispersion and a few lower scores. For the total score, the experimental group again shows a higher median and lower variability than the control group.

Expert interviews provided additional support for the statistical results. Experts noted that, under identical time constraints and scoring criteria, the experimental group produced works with more consistent style, finer detail, and stronger overall visual presentation, consistent with their higher scores in the Technicality and Expressiveness dimensions. The rapid generation and iteration features of GenAI allowed students to refine their visual solutions repeatedly within a limited time, improving composition, color coordination, and overall completeness. In contrast, iterative processes in the control group were more constrained, and some works showed greater variation in precision and stylistic consistency.

To assess the reliability of expert ratings, a two-way random-effects absolute agreement Intraclass Correlation Coefficient (ICC) was calculated based on ratings from six experts (see Table 14). The results indicate excellent mean-rating consistency (ICC(2, k) = 0.924, 95% CI [0.847, 0.947]) and acceptable single-rater consistency (ICC(2, 1) = 0.670), demonstrating that the evaluation results are reliable.

Overall, the expert-rating results were consistent and in trend with the structural model analysis, providing external convergent evidence that GenAI-integrated instruction contributes to improving the quality of learning outcomes. It should be emphasized that expert ratings serve as an external evaluation indicator of learning outputs and are intended to supplement the characterization of performance differences under different instructional models, rather than to test specific causal pathways.

### 4.7. Analysis of Lecturer and Learner Interview Results

To provide additional insights into learners’ and lecturers’ experiences in the GenAI-integrated course, structured interviews were conducted after the instructional experiment. The interview design and coding procedures are described in Section 3.5.3.

The results suggest that lecturers and students shared broadly similar perceptions of learning experiences, motivation, and instructional integration, while some differences were observed in instructional control, creative flexibility, and evaluation focus. These findings provide contextual insights that complement the quantitative results.

#### 4.7.1. Learning Experience and Changes in Cognitive Load

Lecturers and students reported that the use of GenAI reduced time spent on task execution and improved efficiency during the concept-development stage. Several students noted that AI-generated images provided intuitive visual references, helping them enter the creative process more quickly. Lecturers also observed that, as students became more familiar with GenAI tools, the effort required for information retrieval, material generation, and repeated trial-and-error gradually decreased, supporting more efficient task progress.

At the same time, participants described an initial adaptation phase. Lecturers noted that students initially needed additional effort to understand how to use GenAI tools, which temporarily increased cognitive load. However, this burden decreased as familiarity improved (Cierniak et al., 2009). Students in the experimental group appeared to complete tasks more efficiently, while those in the control group spent more time on manual sketching and exploration. These observations suggest that learners’ experiences with GenAI followed a stage-related pattern, with higher initial effort followed by gradual reduction as proficiency increased.

Lecturers also pointed out that without clear guidance, the convenience of GenAI might reduce the depth of students’ thinking. When task structures and evaluation criteria were clearly defined, students shifted their focus from tool operation to conceptual judgment and solution selection (Alipour, 2020). Overall, these findings provide contextual insights that complement quantitative results, particularly in illustrating variations in learning experiences and perceived effort.

#### 4.7.2. Learning Motivation and Emotional Feedback

Interview results suggest that the use of GenAI enhanced students’ interest in learning and their level of classroom participation. Students reported that the diversity of AI-generated outputs expanded their exploratory space, making them more willing to try different design directions. Lecturers also observed higher levels of participation in discussions and presentations, contributing to a more positive learning atmosphere.

At the same time, some lecturers noted that when students relied heavily on AI-generated results, a few experienced fluctuations in their sense of ownership over their work, which could influence their self-evaluation. Overall, these findings suggest that GenAI may support learning motivation by increasing interest and the perceived relevance of course content, while its influence on self-efficacy appears to vary depending on context. These results provide additional context for understanding patterns observed in the quantitative analysis.

#### 4.7.3. Perceptions of Integrated Teaching Alignment (ITA)

Interview results suggest that the introduction of GenAI influenced classroom organization and teacher–student interaction. Lecturers reported that AI tools improved the immediacy of feedback and increased the frequency of interaction, while students described a more collaborative and engaging classroom experience when exploring GenAI together with their lecturers.

In addition, some lecturers noted that as the completeness of AI-generated outputs improved, assessment methods based solely on final products became less sufficient for capturing students’ learning processes. As a result, greater attention was placed on students’ tool-use strategies and the articulation of their design ideas.

Overall, these findings provide classroom-level insights into how instructional integration was perceived in the GenAI-integrated course, and offer contextual support for understanding patterns observed in the quantitative results.

## 5. Discussion

### 5.1. Main Findings

Based on the six-week GenAI-integrated illustration course, the results show that integrating generative AI does not directly reduce learners’ cognitive load but is associated with changes in the relationships among key learning variables. The findings highlight the important role of perceived instructional coherence in shaping learning satisfaction in GenAI-supported contexts. Overall, the results indicate higher learning engagement, more positive learning experiences, and improved learning outcomes.

First, for learning outcomes, the experimental group outperformed the control group across multiple dimensions of expert evaluation, indicating an advantage of GenAI-integrated instruction in supporting design performance. This is consistent with the higher levels of learning satisfaction and perceived course relevance reported in the questionnaires, suggesting that the use of GenAI is associated with improvements in both learning processes and outcomes.

Second, regarding the structural relationships among variables, the SEM results show that traditional pathways related to cognitive load and attention had weaker predictive roles under GenAI-supported conditions. In contrast, learners’ perceptions of instructional integration (ITA) emerged as a more stable factor associated with learning satisfaction. Multi-group analysis and path comparisons further suggest that relationships among cognitive and motivational variables vary across instructional contexts. These findings indicate that GenAI-integrated instruction is associated with context-dependent variations rather than simply strengthening existing pathways.

Third, motivation-related results show that perceived course relevance contributes to learning confidence but is not directly associated with learning satisfaction. This suggests that the role of motivation depends on the broader instructional context. Combined with interview findings, the alignment among teacher guidance, task structure, and technological support emerges as an important contextual factor influencing the stability of the learning experience.

In summary, the findings suggest that, in GenAI-integrated design education, learning effectiveness is not primarily associated with a single cognitive-load or motivational variable. Instead, it is more closely related to the coordination among instructional structure, technological support, and learning experience.

### 5.2. Patterns of Relationships Among Cognitive Load, Learning Motivation, and Instructional Integration in GenAI-Integrated Instruction

The empirical results from GenAI-integrated design instruction suggest that learning outcomes cannot be explained by a simple linear effect of any single psychological variable. Instead, they reflect changes in the overall pattern of relationships among multiple variables. By synthesizing results from the structural equation model and multi-group comparisons, learners’ perceptions of instructional coherence emerge as a key factor in the model. This pattern is better understood as a context-specific association within the current instructional environment rather than as evidence of a broader directional shift.

Further analysis shows that, in the GenAI-integrated visual design course examined in this study, some cognitive-load-related paths derived from CLT were not statistically supported. This does not imply that cognitive load theory is invalid. Instead, learning satisfaction was more strongly associated with perceived instructional alignment and selected motivational factors than with the cognitive-load-related paths in the model. This finding offers a context-specific perspective on the role of CLT-related variables in intelligent instructional settings. However, these patterns should be interpreted cautiously and require further verification through longitudinal or controlled experimental research.

It should be noted that the indirect effects identified in this study are exploratory and supplementary in nature and are therefore not interpreted as stable or confirmatory mediation mechanisms.

#### 5.2.1. Contextual Patterns of Cognitive Load and Their Limited Associations in the Model

From the perspective of CLT, prior research has generally emphasized the differentiated roles of intrinsic load, extraneous load, and germane load in the learning process. However, in the GenAI-integrated instructional context examined in this study, the predictive effects of cognitive-load-related paths were not consistently supported in the overall model, and neither were the effects of intrinsic and extraneous load on attention, nor the effect of germane load on course relevance, which formed statistically significant paths within the full sample.

This result does not imply that cognitive load theory becomes invalid in GenAI-supported instruction. Rather, it suggests that the relationships involving cognitive load may vary across specific instructional contexts. In environments where GenAI provides instant generation, multimodal presentation, and automated processes, learners’ experiences may involve different patterns of information processing and resource allocation (Skulmowski, 2023). Under such conditions, the explanatory role of cognitive-load-related variables for learning experience and outcomes may not be reflected through simple linear pathways.

At the same time, descriptive statistics show that the experimental group reported higher mean scores on intrinsic, extraneous, and germane load, along with advantages in learning motivation and outcome performance. This pattern does not support a straightforward interpretation that higher cognitive load leads to better learning outcomes. Instead, it suggests that, in GenAI-integrated instruction, cognitive load indicators may reflect multiple aspects of the learning process, including tool use, task complexity, and interaction with AI-supported systems.

Further analysis showed that learning satisfaction was more strongly associated with learners’ perceived instructional alignment (ITA) than with cognitive-load-related variables under the conditions of this study. These findings suggest that, within GenAI-integrated instruction, the relationships among cognitive load, motivation, and learning outcomes may differ from those observed in more traditional instructional settings. However, these patterns should be interpreted cautiously and require further validation in future research.

#### 5.2.2. Learning Motivation in Context: The Role of Relevance and Its Associations with Confidence and Satisfaction

Within the ARCS framework, this study found that course relevance remains a key factor in GenAI-supported instruction, and its positive effect on learning confidence was supported in the overall model. This suggests that when learning tasks are clearly connected to learners’ goals, interests, or real-world needs, GenAI does not weaken learning motivation but may instead strengthen the process through which motivation develops (Chien et al., 2025).

However, further analysis showed that course relevance and learning confidence did not directly and consistently predict learning satisfaction. In both the full sample and the multi-group comparisons, the effects of motivational variables on learning outcomes exhibited clear context dependence. This suggests that, in GenAI-integrated instructional environments, learning motivation remains a necessary, but no longer a sufficient condition for explaining differences in learning experience and outcomes.

This finding helps to explain why some motivation-related paths perform inconsistently across instructional contexts: when alignment is lacking among instructional structure, task organization, and technological support, even learners who maintain strong interest or confidence in the content may not necessarily translate these positive experiences into stable learning satisfaction. Therefore, the motivational components in the ARCS model may operate in conjunction with broader instructional conditions, rather than consistently functioning as independent predictors of learning outcomes.

#### 5.2.3. The Explanatory Role of Integrated Teaching Alignment (ITA) and Its Position in the Model

Compared with cognitive-load and learning motivation paths, ITA showed a more stable and significant association in this study. The structural model results indicate that ITA has a direct positive association with learning satisfaction, and multi-group analyses further show that this relationship remains relatively consistent across different instructional contexts.

In this study, learning satisfaction is more consistently associated with learners’ perceived instructional coherence (ITA) than with individual cognitive-load or motivational variables. When teacher guidance, task design, and GenAI tool support are well-aligned, learners are more likely to perceive technology as a natural part of the learning process rather than an additional burden, contributing to a more stable and positive learning experience.

From an explanatory perspective, the relatively stable role of ITA suggests its importance in shaping the learning experience. However, this role should be understood as offering an integrative perspective on the relationships among CLT and ARCS variables rather than replacing existing theoretical explanations. In other words, ITA may help explain how cognitive load and learning motivation interact within GenAI instructional contexts. Nevertheless, its broader applicability requires further testing across different disciplines and research designs.

Taken together, these findings suggest that GenAI-integrated design instruction does not simply strengthen or weaken any single component of existing learning theories. Instead, the results highlight ITA as an integrative factor linking cognitive and motivational pathways within the instructional context. This provides a useful perspective for understanding how relationships among learning variables may change in GenAI-supported environments and offers practical implications for design education.

### 5.3. The Theoretical Significance of Integrated Teaching Alignment (ITA) and Its Implications for Design Education

Building on the combined perspective of CLT and ARCS, this study highlights the potential role of GenAI in design education. The integration of GenAI may be associated with variations in cognitive and motivational processes, as well as changes in instructional structure, lecturer roles, and evaluation approaches (Lu et al., 2024). The educational implications of GenAI can be understood from an integrated perspective.

Based on the preceding analysis, ITA should not be understood as a simple combination of instructional elements. Instead, in GenAI-integrated contexts, it can be interpreted as an integrative perspective for understanding how key elements of the learning process are organized. The results indicate that ITA shows a stable association with learning satisfaction across instructional contexts and provides a useful lens for examining instructional design in GenAI-supported environments.

At the level of instructional structure, GenAI-integrated instruction can be viewed as moving beyond simple “tool insertion” toward a more coordinated design of tasks, tools, and feedback. When GenAI is used only as an auxiliary tool, its association with the learning experience may be limited. In contrast, stronger perceptions of integration appear when it is embedded within a coherent task structure. In this sense, GenAI can be understood as part of the learning workflow rather than as a single-stage tool.

At the level of teacher roles, the findings suggest a greater emphasis on process guidance and evaluative support. Learning satisfaction appears to be more closely related to learners’ perceived coherence within the instructional process than to cognitive investment or motivational intensity alone (Walter, 2024). Teachers, therefore, play an important role in structuring tasks, guiding tool use, and providing formative feedback.

At the level of assessment, the results highlight the importance of learning processes and strategies. Evaluation based solely on final outputs may not fully capture learning quality. Instead, greater attention may be given to how learners understand tasks, use tools, and make design decisions, with formative assessment complementing summative evaluation (Pan et al., 2025).

Taken together, ITA offers an integrative perspective for understanding how learning factors relate within GenAI-supported instruction. It does not replace existing theories but provides a useful perspective for understanding how cognitive and motivational elements are coordinated within GenAI-supported instructional structures.

### 5.4. Comparison with Prior Research and Theoretical Extension

Based on the above analysis, the findings of this study can be situated within the broader context of GenAI and design education research. Overall, the results are generally consistent with prior studies on GenAI-supported learning and design education. In particular, the positive associations among learning motivation, perceived relevance, and learning outcomes align with existing evidence that motivational factors play an important role in technology-enhanced learning environments. In addition, the integration of GenAI tools into instructional processes appears to support learners’ engagement and creative performance, consistent with previous findings in design education contexts.

At the same time, the findings also highlight certain differences from prior interpretations. Rather than attributing learning outcomes primarily to cognitive load or motivational factors alone, the results suggest that learners perceived coherence among instructional structure, technological support, and learning processes (i.e., ITA) is consistently associated with learning satisfaction across contexts.

These findings indicate that, in GenAI-supported learning environments, relationships among learning factors may be better understood from the perspective of instructional coherence and integration. In the present study, this was reflected in the relatively stable association between ITA and learning satisfaction across analyses. In this sense, ITA can be viewed as a complementary perspective for examining how cognitive and motivational elements are related within instructional contexts, rather than as a replacement for existing theoretical frameworks.

Overall, these findings provide a context-specific perspective on how learning processes are organized in GenAI-supported design education and offer implications for understanding the role of instructional structure in technology-integrated learning environments.

## 6. Conclusions

This study examined the integration of GenAI into a visual design course based on CLT and the ARCS model and implemented a design-thinking-oriented teaching experiment. Using a teaching experiment and PLS-SEM analysis, the results suggest that, within the present course context, GenAI-integrated instruction is associated with improved learning experiences and differences in the relationships among learning-related variables.

The results indicate that, under the current model specification, cognitive-load-related paths and several motivation-related paths do not consistently predict learning satisfaction. In contrast, learners’ perception of ITA, referring to the overall coherence among instructional goals, task flow, teacher guidance, and GenAI support, shows a more stable relationship with learning satisfaction in the structural model. These findings suggest that alignment between instructional structure and technological support may be more closely related to learners’ satisfaction than individual cognitive load or motivational variables.

From a theoretical perspective, this study highlights the contextual characteristics of learning processes in GenAI-supported instructional environments. By introducing ITA as an integrative perspective, it provides a framework for understanding how CLT- and ARCS-related factors interact under intelligent instructional conditions. This perspective does not replace CLT or ARCS; rather, it complements these theories by helping explain why some expected relationships appear less stable in GenAI-integrated contexts. In practical terms, the findings suggest that the effective use of GenAI in design education depends less on isolated tool use and more on coherent instructional design. Learning experiences are more likely to benefit when task structure, teacher guidance, and technological support are aligned throughout the instructional process.

Overall, these findings provide empirical responses to the research questions proposed in Section 1. The results suggest that GenAI-integrated instruction is associated with variations in learners’ cognitive load and learning motivation (RQ1), while the combined CLT–ARCS framework offers a perspective for understanding the relationships among cognition, motivation, and learning outcomes in AI-supported learning environments (RQ2). In addition, the results highlight the moderating role of Integrated Teaching Alignment (ITA) in shaping learning satisfaction (RQ3), and indicate that GenAI-integrated instruction may show advantages over traditional teaching approaches for creative performance and learning experience (RQ4). Furthermore, the structural model results provide partial support for the proposed research hypotheses.

Despite the progress made in the theoretical analysis and empirical validation of the model, several limitations should be acknowledged. First, the sample size was relatively small (N = 120). Although it meets the basic requirements for exploratory PLS-SEM, it may limit the stability and generalizability of some path estimates. Second, the study was conducted in a visual design course within a specific cultural and institutional context, which may limit the applicability of the findings to other disciplines and educational settings. Third, the instructional intervention lasted six weeks and may not capture longer-term learning effects. Future research should, therefore, examine GenAI-integrated instruction with larger and more diverse samples across multiple disciplines and adopt longitudinal designs to further explore its influence on learning processes and outcomes.

## Figures and Tables

**Table 1:** Baseline equivalence test between experimental and control groups.

Baseline Indicators	Control (*n* = 60)	Experimental (*n* = 60)	Test	*p*	Effect Size
Prior GenAI use (Yes/No)	31/29	32/28	χ^2^(1) = 0.071	0.791	φ = 0.034
GenAI use frequency (1–5)	2.10 ± 0.85	2.17 ± 0.88	t(118) = 0.313	0.755	d = 0.081
Prompt skill self-rating (1–5)	2.05 ± 0.80	2.12 ± 0.78	t(118) = 0.343	0.733	d = 0.089
Baseline design task score (0–100)	74.6 ± 6.8	75.4 ± 7.1	t(118) = 0.446	0.657	d = 0.115
Baseline motivation score (1–5)	3.42 ± 0.54	3.47 ± 0.56	t(118) = 0.352	0.726	d = 0.091

Note. Continuous variables are reported as Mean ± SD and compared using independent-sample *t* tests. Categorical variables were compared using χ^2^ tests. Effect sizes are reported as Cohen’s d and φ. All baseline differences were non-significant with small-effect sizes.

**Table 2:** Comparison between the control group and the experimental group.

Comparison Dimension	Control Group (Traditional Teaching Model)	Experimental Group (Integrated Teaching Model)
Teaching Objectives	Emphasizes skill training and task completion; GenAI is used only as an auxiliary tool.	Emphasizes creativity and expression driven by design thinking, as well as human–AI collaboration.
Tool Integration Method	GenAI tools are used sporadically (e.g., sketch generation, style reference); manual drawing and conventional digital software remain dominant.	GenAI is embedded throughout all five stages, forming a complete closed loop of the “task chain + tool chain.”
Task Structure	Primarily based on independent assignments; task chains are short and lack progressive iteration.	Six-week modular task chain covering empathy–definition–ideation–prototype–testing; emphasizes group collaboration and iterative cycles.
Teaching Methods	Lecturer-led instruction and case analysis; learners are passive recipients.	Workshop-based, guided by design thinking; learners engage in active exploration, reflection, and human–AI co-creation.
Evaluation Mechanism	Focuses mainly on final outcomes; lacks multidimensional and process-oriented feedback.	Combines both outcomes and processes; integrates CLT and ARCS models; includes expert evaluation, peer and lecturer co-assessment, and motivational analysis.

**Table 3:** Expert evaluation dimensions and descriptions.

Dimension	Evaluation Focus	Detailed Description
Creativity	Novelty of concept and visual expression	Evaluates the uniqueness and degree of innovation in conceptual thinking, narrative logic, and visual language.
Applicability	Alignment between task and context	Emphasizes how well the work aligns with course objectives, application scenarios, and the needs of target audiences.
Technicality	Technical and craftsmanship proficiency	Assesses the quality of composition completeness, color control, stylistic consistency, and detail refinement.
Expressiveness	Esthetic and emotional communication	Focuses on the artistic appeal, emotional atmosphere, and visual storytelling effect of the work, highlighting esthetic and emotional depth.
Completeness	Logical coherence and overall integrity	Evaluates the overall coherence, adequacy of information, and rationality of design explanations, including midterm development, extended application, and project presentation.

**Table 4:** Description of research variables and measurement dimensions.

Model	Dimension	Core Measurement Focus	Example Item	Reference
CLT	IL	Task complexity and learning difficulty	“The course tasks are too complex for me.”	Sweller (1988) Sweller et al. (1998, 2011)
	EL	Distraction caused by tools or instructional design	“I experienced unnecessary operational interference when using the tools.”	
	GL	Cognitive engagement facilitating knowledge construction	“The course activities helped me transform knowledge into practical creation.”	
ARCS	AT	Whether the course captures and sustains interest and attention	“The course activities were able to attract my attention.”	Keller (1987, 2009)
	RE	Alignment between learning content and personal goals	“The course content is highly relevant to my learning needs.”	
	CF	Self-efficacy in completing tasks	“I am confident that I can complete the course assignments.”	
	SA	Overall satisfaction with learning outcomes and experience	“I am satisfied with the overall experience of this course.”	
TPACK Constructivism	ITA	Learners’ perception of process clarity, feedback loop, and instructional support	“The course steps were clearly structured, and each stage had specific tasks.”	Davis (1989); Taylor et al. (1997)

**Table 5:** Lecturer interview themes, questions, and research objectives.

Theme	Lecturer Interview Questions	Research Objective
Teaching Cognition and Preparation	(1) When designing the two types of courses (traditional vs. GenAI-integrated), what were your main considerations and differences in thinking? (2) After introducing GenAI, what changes occurred in your teaching preparation and classroom management? (3) Compared with traditional teaching, at which stages of the GenAI-integrated course did you observe the most significant differences?	To analyze how GenAI integration influences lecturers’ cognition and TPACK structure.
Teaching Implementation Experience	(4) How do you balance the relationship between AI-generated results and students’ originality?	To explore lecturers’ strategies for facilitating human–AI collaboration and creative development.
Learner Response	(5) What differences did you observe in learners’ motivation and engagement when using GenAI? (6) Does GenAI reduce learners’ cognitive load or improve task efficiency?	To verify CLT and ARCS mechanisms from the lecturers’ perspective.
Teaching Reflection and Challenges	(7) What aspects of teaching were the most challenging for you (e.g., technical mastery, classroom management, or evaluation criteria)? (8) Has the introduction of GenAI changed your understanding of the “lecturer’s role”?	To reveal issues related to lecturer role transformation and the alignment of teaching strategies.
Teaching Optimization and Future Outlook	(9) If you were to redesign the course, how would you modify the use of GenAI? (10) What do you think will be GenAI’s position and role in the future of design education?	To gather lecturers’ suggestions for instructional improvement and educational value assessment.

**Table 6:** Experimental group learner interview themes, questions, and research objectives.

Theme	Learner Interview Questions	Research Objective
Learning Experience and Motivation	(1) How did your learning experience in the GenAI course differ from that in the traditional course? (2) Which instructional approach better stimulated your learning interest?	To verify motivational differences (ARCS model).
Task and Cognitive Load	(3) In the GenAI course, did you find the tasks easier or more complex? Why? (4) How did AI tools assist or constrain your thinking and creative process?	To verify changes in cognitive load (CLT).
Creativity and Learning Outcomes	(5) Do you think GenAI helped you enhance your creative expression? (6) Were you satisfied with your work upon completion?	To explore learning effectiveness and satisfaction.
Teacher–Learner Interaction and Collaboration	(7) How did the lecturer’s guidance differ in the GenAI course compared with the traditional one? (8) Did you find communication within your group collaboration smoother?	To compare learning experiences in social constructivist contexts.
Teaching Optimization and Future Outlook	(9) If you were to redesign the course, how would you modify the use of GenAI? (10) What do you think will be GenAI’s position and role in the future of design education?	To gather lecturers’ suggestions for instructional improvement and educational value assessment.

**Table 7:** Group comparison of descriptive statistics.

	Experimental (*n* = 60) M	Experimental SD	Control (*n* = 60) M	Control SD
IL	3.7	1.211	3	1.111
EL	3.778	1.08	2.822	1.009
GL	3.744	1.196	3.133	1.237
AT	3.7	1.095	3.089	1.148
RE	3.778	0.948	3.156	1.082
CF	3.456	0.981	3.344	1.077
SA	3.767	0.798	3.378	1.117
ITA	3.844	1.038	3.544	1.255

**Table 8:** Reliability and convergent validity of measurement constructs.

Construct	Cronbach’s Alpha	Composite Reliability (rho_C)	AVE
AT	0.951	0.966	0.905
CF	0.920	0.950	0.862
EL	0.954	0.939	0.642
GL	0.961	0.973	0.924
IL	0.969	0.950	0.863
ITA	0.918	0.949	0.861
RE	0.779	0.870	0.705
SA	0.874	0.921	0.796

**Table 9:** Heterotrait–Monotrait Ratio (HTMT) for discriminant validity.

Construct	AT	CF	EL	GL	IL	ITA	RE	SA
CF	0.185							
EL	0.103	0.124						
GL	0.138	0.117	0.242					
IL	0.052	0.222	0.075	0.050				
ITA	0.100	0.144	0.194	0.107	0.198			
RE	0.194	0.405	0.122	0.177	0.183	0.133		
SA	0.397	0.479	0.151	0.113	0.079	0.509	0.404	
ITA × RE	0.166	0.086	0.087	0.071	0.171	0.264	0.201	0.177

Note. All HTMT values are below the conservative threshold of 0.85, indicating adequate discriminant validity among constructs.

**Table 10:** Results of Compositional Invariance Assessment (MICOM Step 2).

Construct	Original Correlation (c)	5% Quantile (c_0.05_)	Permutation *p*-Value
AT	0.993	0.955	0.281
CF	0.896	0.831	0.089
EL	0.350	0.195	0.063
GL	0.992	0.934	0.192
IL	0.934	0.432	0.158
ITA	1.000	0.986	0.976
RE	0.982	0.852	0.524
SA	0.999	0.993	0.651

Note: All permutation *p*-values exceed 0.05, suggesting that compositional invariance is established across constructs.

**Table 11:** Multi-group path difference and hypothesis test results.

Hypothesis ID	Path Relationship	Coefficient (Exp)	Coefficient (Ctrl)	Difference	*p*-Value	Verification Result
H1	IL → AT	−0.093	−0.269	0.176	0.626	Group difference not significant
H2	EL → AT	−0.199	0.157	–	0.389	Group difference not significant
H3	GL → RE	0.123	0.336	–	0.037	Group difference significant
H4	AT → RE	−0.108	−0.345	0.236	0.005	Group difference significant (opposite direction)
H5	RE → CF	0.383	−0.062	0.445	0.238	Group difference not significant
H6	RE → SA	0.289	−0.352	0.641	0.009	Group difference significant
H7	CF → SA	0.245	0.583	–	0.151	Directional difference exists between groups
H8	ITA × RE → SA	0.327	0.357	−0.030	0.916	Group difference not significant

**Table 12:** Supplementary comparison of selected indirect effects across groups.

Indirect Path	Coefficient (Exp)	Coefficient (Ctrl)	Difference	*p*-Value	Conclusion
AT → RE → SA	−0.031	0.121	−0.153	0.005	Group difference significant
GL → RE → SA	0.035	−0.118	0.154	0.037	Group difference significant
IL → AT → RE → SA	0.003	−0.033	0.035	0.007	Group difference significant
Other indirect paths	—	—	—	*p* > 0.05	Group difference not significant (*p* > 0.05)

**Table 13:** Expert evaluation results of student works.

Work_ID	Creativity (M)	Applicability (M)	Technicality (M)	Expressiveness (M)	Completeness (M)	Score (M)
W01/Exp	88.50	85.00	87.67	89.17	86.67	87.40
W02/Exp	87.50	87.50	87.50	87.33	88.33	87.63
W03/Exp	89.00	83.17	86.67	87.00	87.00	86.57
W04/Exp	86.33	81.83	80.50	85.83	82.50	83.40
W05/Exp	88.33	85.67	86.00	88.83	86.50	87.07
W06/Exp	87.00	86.00	87.50	87.50	87.00	87.00
W07/Exp	89.33	84.67	87.50	87.83	88.00	87.47
W08/Exp	87.67	87.67	87.50	87.50	88.67	87.80
W09/Exp	89.83	86.50	89.00	89.17	88.17	88.53
W10/Exp	82.17	79.83	84.00	83.67	81.83	82.30
W11/Exp	88.33	85.00	85.33	88.83	86.00	86.70
W12/Exp	89.17	84.83	85.67	87.83	88.17	87.13
W13/Ctrl	80.00	83.50	79.00	80.83	81.17	80.90
W14/Ctrl	73.50	76.67	74.33	76.00	77.17	75.53
W15/Ctrl	74.67	83.50	74.83	75.33	78.83	77.43
W16/Ctrl	84.50	84.33	79.67	83.67	83.33	83.10
W17/Ctrl	81.00	82.50	78.67	80.50	80.17	80.57
W18/Ctrl	81.50	79.00	76.50	80.17	79.33	79.30
W19/Ctrl	85.33	85.83	85.67	85.33	85.00	85.43
W20/Ctrl	76.67	78.00	73.00	75.50	78.17	76.27
W21/Ctrl	83.83	84.17	83.00	84.17	82.50	83.53
W22/Ctrl	77.67	81.00	78.17	78.50	79.67	79.00
W23/Ctrl	80.17	79.67	77.50	82.00	80.67	80.00
W24/Ctrl	78.50	82.33	75.83	76.00	78.33	78.20

**Table 14:** Inter-rater reliability of expert evaluations calculated using a two-way random-effects ICC with absolute agreement.

Indicator	Estimate	95% Confidence Interval (CI)	Interpretation (Koo & Li, 2016)
ICC (2, 1) (Single-rater reliability)	0.670	0.476–0.748	Moderate–Good
ICC (2, k) (Mean-rating reliability of 6 raters)	0.924	0.847–0.947	Excellent reliability

## Data Availability

Data are contained within the article.

## Abbreviations

The following abbreviations are used in this manuscript:

CLT	Cognitive load theory
ITA	Integrated Teaching Alignment
SDG	Sustainable Development Goal
IL	Intrinsic load
EL	Extraneous load
GL	Germane load
AT	Attention
RE	Relevance
CF	Confidence
SA	Satisfaction
GenAI	Generative artificial intelligence
ICC	Intraclass Correlation Coefficient
ARCS	Attention, Relevance, Confidence, Satisfaction
PLS-SEM	Partial Least Squares Structural Equation Modeling
TPACK	Technological Pedagogical Content Knowledge
